# Finding Effective Adjustment Levels for Upper Limb Exergames: Focus Group Study With Children With Physical Disabilities

**DOI:** 10.2196/36110

**Published:** 2023-01-13

**Authors:** Martina Eckert, Beatriz Domingo Soria, Noelia Terroso Gil

**Affiliations:** 1 Group of Acoustics and Multimedia Applications Department ot Audiovisual Engineering Universidad Politécnica de Madrid Madrid Spain; 2 Department of Physiotherapy Primary School Centro de Educación Infantil y Primaria Pinar de San José Madrid Spain

**Keywords:** rehabilitation, physical therapy, cerebral palsy, obstetric brachial plexus palsy, serious games, exergames, Kinect

## Abstract

**Background:**

We developed the Blexer system consisting of a database and a web interface for therapists that can host different types of adaptive and personally configurable virtual reality exergames based on Kinect (Microsoft Corp) motion capture to provide entertaining exercises for children with motor disabilities. It allows for parameter adjustment and the monitoring of results remotely, thereby providing a useful tool to complement traditional physical therapy sessions with home exercises.

**Objective:**

The aim of this study was to observe the motor benefits achieved through the use of a video exergame and the importance and implications of correctly setting the game’s difficulty parameters.

**Methods:**

This was an observational case study of 6 children with different physical disabilities receiving physical therapy at school combined with the use of a fully configurable exergame under research that forms a part of the Blexer environment. The game integrates 4 repeatedly appearing upper limb exercises with individually adjustable difficulties (intermittent arm rising, arm forward and backward movement, rising and holding of one arm, and trunk control in all directions). The outcomes were 3 assessments of 2 efficacy measures: Box and Block Test and Jebsen Taylor Hand Function Test.

**Results:**

A total of 5 children with cerebral palsy (mean 8.4, SD 2.7 years; Gross Motor Function Classification II—2/5, 40%; III—2/5, 40%; and IV—1/5, 20%) and 1 child with obstetric brachial plexus palsy (aged 8 years; Mallet Classification III) received between 8 and 11 sessions of training (10-20 minutes per session), depending on age, motivation, and fatigue. Significant associations were observed between game parameter settings and improvements in motor function, on the one hand, and between the type of improvement and disability severity, on the other: with adjusted game parameters *goal* and *time* in the range of 70% to 100%, only less affected children improved in the Box and Block Test (+11 blocks vs −1 block), and more affected children improved more in the Jebsen Taylor Hand Function Test (+90 seconds vs +27 seconds).

**Conclusions:**

When defining the difficulty parameters for an exergame, we suggest a classification in levels ranging from *very easy* to *very hard*. For practical use, we suggest setting the difficulty for the player to an *easy* or *medium* level rather than high-commitment goals, as this leads to a longer playtime with more fun and, therefore, seems to improve the results of the game and, consequently, mobility.

## Introduction

### Background

Cerebral palsy (CP) and obstetric brachial plexus palsy (OBPP) are 2 of the pathologies that most frequently cause problems in the upper limbs in children and adolescents. According to the International Classification of Functioning, Disability, and Health (ICF), these problems affect corporal structure and function, limiting, in many cases, activities of daily living [1]. CP is considered a global disorder because of a permanent and nonprogressive lesion in the brain, before its development is complete, producing alterations in tone, posture, and movement, resulting in functional limitations and other associated alterations [2,3]. OBPP is generally caused by an elongation of the brachial plexus during childbirth, although it can also have an intrauterine, genetic, or postural origin. Alterations range from nerve conduction impairment to total rupture of anatomical structures [4,5]. Common problems of both pathologies are muscular atrophy and weakness, imbalance, alteration of the coordination of different muscle groups, secondary joint pathology, and decreased general use of the arms and hands in daily activities [6,7].

Regarding the different conservative treatments for both pathologies, we found that different systematic reviews proposed repetitive, challenging, high-intensity, and motivational interventions [5,6,8,9].

When analyzing the interventions that are considered effective in CP, we found that training with video games or virtual reality (VR) is among the interventions for which there is promising evidence suggesting possible effectiveness [8]. In OBPP, VR associated with other therapeutic strategies is also considered significantly more effective than conventional physical therapy programs [4,10]. Therefore, serious games and VR are considered promising interventions in both conditions.

Systematic reviews that analyzed the effectiveness and clinical utility of VR interventions indicated that motion capture devices have beneficial effects in the rehabilitation of patients with neurological disorders, achieving high levels of compliance, motivation, and commitment that can help improve one or more levels of ICF. The greatest benefits were found in domain 1 of the ICF, being effective in improving upper extremity function, hand coordination, balance function, gait, and postural control. In addition, benefits are associated with aspects such as participation in the community and improvements in psychological and cognitive functions [5,9,11-13].

The benefit of video games is that they offer immediate feedback and rewards that stimulate the brain so that the user wants to play more. Providing challenging and fun environments that keep the player motivated increases the number of repetitions of certain movements compared with conventional therapy. Thus, neuroplastic changes and cortical reorganization, which could produce improvements in performance, are elicited [14,15]. The use of these types of games encourages participants to direct their attention to an external focus, which helps them to learn faster and more accurately than requests from an internal focus [16].

As for motion-controlled video games, most studies use Kinect devices (Microsoft Corp) along with commercial video games, which provide accessibility and portability and are low cost. However, these video games do not address the specific needs of patients who sometimes cannot perform the required movements quickly or efficiently enough to succeed during play. Therefore, different reviews point to the advantages of specifically designed games that provide appropriate challenges and adapt to the player’s speed and quality of movement, thus avoiding boredom and frustration. However, the negative aspect is that commercially available mainstream video games cannot be personalized and that there are still very few offers for therapeutic video games, which are quite expensive [11-13,17-20].

Some commercial solutions we found are the MIRA software platform (MIRA Rehab Limited), [21], VirtualRehab (Evolv) [22], and REHABILITY (Imaginary SL) [23], all of which seem quite complete, but it is not clear from the information provided on the web pages how and to what extent the different exercises involved in the games are configurable for each user.

The common opinion of researchers is that games should complement therapy with both options, accompanied by a professional or used at home [9,12,24]. In any case, it is recommended that computer-based training be followed by the practice of activities in the physical environment to ensure adequate perceptual adjustment and the transfer of the benefits of training to activities of daily living performed in a real environment [24].

### Objectives

The purpose of this study was to observe how much influence the adjustment of the games’ objectives has on the child’s performance in upper limb movements. We suppose that there is a small range of adjustments that ensures that the challenge for the child is neither too low (leading to boredom) nor too high (leading to frustration). Therefore, we wanted to prove the hypothesis that the optimal settings (of the goal and the time parameters) are those that ensure that the goal can be reached within the given time limit. Furthermore, the aim was to derive some practical adjustment rules from our results that could be useful for our and other similar configurable games.

## Methods

### Therapeutic Video Game Environment

The Blexer system has been described in detail in various previous publications [25,26]. It consists of a database and a web platform that integrates different video games and is accessible only to clinicians. On the user side, a PC with a motion capture camera (Kinect X360, Microsoft Corp) is required. The PC hosts one or more games and a middleware [27] that acts as a communicator among the camera, the database, and the games. It provides the games with motion data, downloads the game settings established by the clinician, and uploads the users’ results after each play to the database.

The game used in this study, Phiby's Adventures (version 1), is a semi-3D third-person video game extensively described in the study by Eckert et al [26]. The main character, Phiby, is a small amphibian that must be helped to get out of a valley, which is represented by a modular 2D map with 16 × 8 cells. The cells are connected through different obstacles such as rivers, trees, lakes, or trunks that Phiby must pass while finding its way out of the valley. Each obstacle is an independent exercise scene (refer to the description provided in Table 1) that needs arm and trunk movements of different intensities to pass through. The logs obtained from chopping the trunks can be used to build bridges or huts. A hut allows the player to rest and to save the game state for resuming from that point in the next session. With the aim of increasing the player’s interactivity and decision-making, they are given an option to freely choose the path to follow and, therefore, take the obstacles they like. By passing through the cells, the areas of the map are gradually discovered and can be visualized through an arm movement. The 4 exercise scenes have adjustable difficulty parameters (objective and time limit), as explained in Table 1. An initial calibration measures the players’ range of movement for adapting the game to their capacities.

**Table 1:** Overview of the 4 exercises inherent in the video game.

Exercise	Description	Abilities to train	Pictures
Chop the wood	Exercise using the affected limb: this exercise provides a specific activity for the use of the affected side in which maximum shoulder flexion is requested based on the initial calibration. The child works on support and shoulder girdle stability to keep the axe elevated until it is loaded as well as on arm speed to lower the axe and split the wood. The amount of wood to split (=number of arm lifts) and the time limit to achieve all can be modified.	Proximal arm movement (shoulder flexion) Movement speed Initiation of use of assisting hand Reaches Endurance Stabilization of the shoulder and shoulder girdle	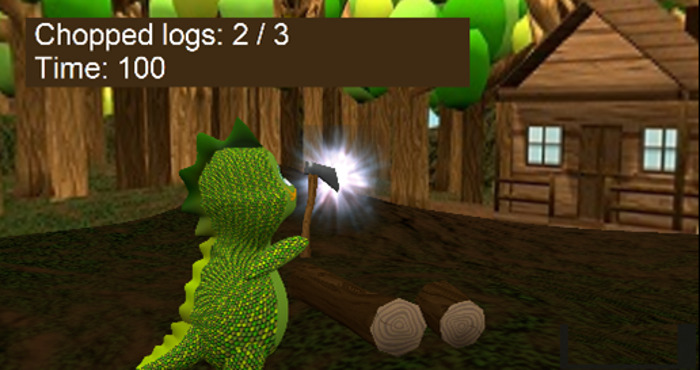
Dive and eat	Trunk control exercise: in this exercise, the objective is to catch some of the pieces of plankton floating in the water. The player controls the direction of the fish with their trunk movements. It favors trunk control and can be performed in a sitting or standing position. The number of planktons to catch and the time limit can be modified.	Trunk control and stability	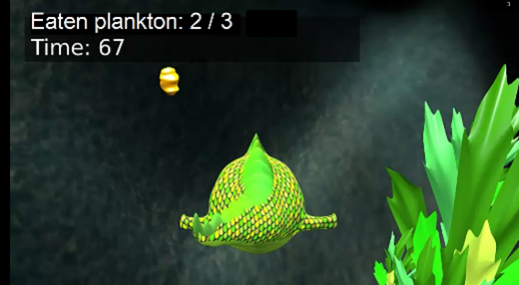
Row the boat	Symmetrical movement exercise of upper extremities: in this exercise, the symmetrical use of both upper limbs is proposed, and the objective is to row a boat until reaching the opposite bank of the river. The speed of the boat depends on the speed of the user’s movements. The distance to travel (=number of arm movements) and the time limit can be adjusted.	Proximal movement of the arm (shoulder flexion-extension) and elbow Movement speed Initiation of use Hand and arm coordination Execution flow of bimanual activities	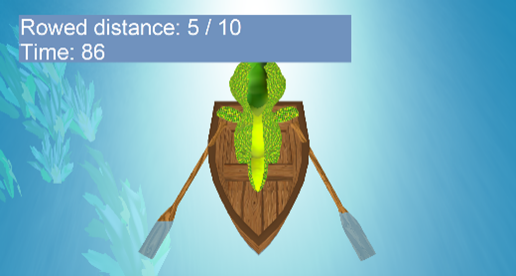
Climb the tree	Alternated movement exercise in upper limb abduction: this exercise involves alternating movements of the arms in abduction, and the objective is to reach the top of a tree to see the valley. The parameters to be modified are the height of the tree (=number of arm movements) and the time limit.	Proximal arm movement (shoulder abduction and flexion) Movement speed Initiation of use Reaches Hand and arm coordination Execution flow of bimanual activities	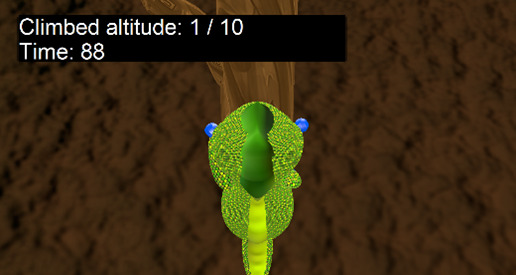

### Study Design and Participants

This case study was jointly driven by the Research Center for Software Technologies and Multimedia Systems for Sustainability of the Universidad Politécnica de Madrid (UPM) and the Public School CEIP (Centro de Educación Infantil y Primaria) Pinar de San José in Madrid. UPM developed the Phiby's Adventures video game and the Blexer-med web platform used in this study. The game is connected to the platform, which allows for the adjustment of the difficulty of the game parameters and the supervision of the results for each participant.

CEIP Pinar de San José is an integrative primary school (5-12 years) with 28 pupils (out of 950) having motor disabilities (13 CP, 2 OBPP). Informed consent was obtained from the parents and children involved in this study.

Inclusion criteria for the study were the following: being a scholar receiving physiotherapy care at the Pinar de San José school, being aged between 5 and 12 years, having a major functional impairment of an upper limb because of CP or OBPP, and being able to understand and follow the game. Exclusion criterion was poor trunk control that prevents activity from being performed in a freely seated position. The children were classified based on their functional level according to the *Gross Motor Function Classification (GMFCS)* [28], *Manual Ability Classification System (MACS)* [28,29], and *Mallet Classification* [30,31].

### Intervention

Initially, several instructional sessions were conducted for 8 weeks to familiarize the children with the dynamics of the game. Then, the intervention took place for 6 weeks, using the game as a part of the usual 45-minute physiotherapy sessions (2 or 3 times a week), for approximately 10 to 20 minutes, according to age, motivation, and fatigue. In the remaining time, they received the usual physiotherapy treatment. They also continued with activities and therapies outside school.

During the instructional sessions conducted before the intervention, all the children played with the same default difficulty adjustments, which were 60 seconds time limit per exercise with the following goals: chopping 60 trunks, climbing up 100 meters, catching 10 planktons diving, and rowing 100 meters. After these sessions, the objectives of each game were adjusted individually for each child according to our subjective observations during training and with the intention to provide affordable challenges.

During the intervention, physiotherapists constantly supervised the children and verbally motivated them. They were allowed to vary their pose (sitting or standing) depending on their abilities and how they felt that day; however, all participants selected their favorite posture once and then always played in the same way and without any physical help from the therapists. Depending on the results observed, that is, considering whether the objectives were achieved and how quickly or whether the children were bored or stressed, the difficulty of each exercise was readjusted after each session to maintain the challenge. In the case of being tired or unmotivated, the goals were lowered, whereas they were raised when improvement or low challenge was observed. The time limit was kept constant at 60 seconds in all exercises. These decisions were based on subjective observations (of movements and mood) as the objective results (time needed for an exercise and score) were not graphically presented on the web at that moment. They could be revised in form of tables, but this was not useful for making instantaneous objective decisions. All the children were able to complete the activity.

### Data Analysis of the Exercise Results and Jebsen Taylor Hand Function Test and Box and Block Test Assessments

The web platform receives the following for each exercise: its difficulty parameters (objective and time limit), the time stamps of entering and leaving, and the score reached. We analyzed the *relative* scores for each type of exercise, that is, the percentage of movements in relation to the objective established by the therapist, and correlated these data with the outcomes of 3 evaluations: previous evaluation (*Vp*) 8 weeks before, initial evaluation (*Vi*) immediately before, and final evaluation (*Vf*) immediately after the intervention. Between Vp and Vi, conventional therapy was performed, and after Vi, the game was used as a part of the therapy sessions for 6 weeks.

The assessments were made using the Box and Block Test (BBT) [32] (counting the number of blocks put from one part of the box to another; the more blocks, the better the result) and the Jebsen Taylor Hand Function Test (JTHFT) [33] (measuring the time required to perform 7 activities; the quicker, the better the result). Both tests have demonstrated reliability, especially for measuring changes that focus on improving hand dexterity and function in children with CP after intensive intervention. The reliability and responsiveness of these tests are better for short evaluation periods [18,19].

Statistical calculations were performed using SPSS software (version 26; IBM Corp). Descriptive statistics were used to summarize baseline characteristics and feasibility data. We used the Levene test to assess the normality of data distribution. Differences of means were calculated and proved by 2-tailed significance tests, and the differences were considered statistically significant at *P*<.05.

### Ethics Approval

This study was approved by the Universidad Politécnica de Madrid (UPM) ethics committee and also complied with the Declaration of Helsinki.

## Results

### Study Design and Participants

For the study, 54% (15/28) of the children aged 5 to 12 years were screened for eligibility among the ones receiving physical therapy support at the Public School CEIP Pinar de San José in Madrid. Out of these, 47% (7/15) met all inclusion criteria and 7% (1/15) had to be excluded because of poor trunk control and inability to maintain stability during play without external aids; so finally, the analysis group was reduced to 6 children (Table 2). The mean age of the participants was 8.3 (SD 2.4) years; 5 (83%) children with CP were aged 8.4 (SD 2.7) years, and 1 (17%) child with OBPP was aged 8 years. We used the following code to identify the participants: *CP/OBPP+age+hand+group.*

The participants were divided into 3 groups based on the results of the functional classifications of GMFCS, MACS, and Mallet (Table 2):

1. Group 1: CP (GMFCS ≥III, MACS ≥III; 3/6, 50% participants)
2. Group 2: CP (GMFCS<III, MACS <III; 2/6, 33% participants)
3. Group 3: OBPP (Mallet III; 1/6, 17% participants)

The participants in group 1 had a lower functional level, with an average age of 7 (SD 2.6) years. The participants in groups 2 and 3 presented a similar profile with higher functional levels in both gross and fine motor skills; therefore, these 2 groups were merged into group 2+3 (average age: 9.7, SD 1.5 years). Thus, 2 homogeneous groups of 3 children each were formed, considering similar functionality and age.

**Table 2:** Details of the participants’ age, sex, diagnosis, affected hand, playing position, motor function ratings, and assigned group.

ID	Age^a^ (years)	Sex	Diagnosis	More affected hand	Play position	Classification				Group
						GMFCS^b^	MACS^c^	Mallet		
CP5L1	5	Female	CP^d^	Left	Seated	IV	III	N/A^e^	1	
CP6R1	6	Male	CP	Right	Seated	III	III	N/A	1	
CP10R1	10	Male	CP	Right	Standing	III	III	N/A	1	
CP10L2	10	Male	CP	Left	Standing	II	I	N/A	2	
CP11R2	11	Female	CP	Right	Standing	II	I	N/A	2	
OBPP8R3	8	Female	OBPP^f^	Right	Standing	N/A	N/A	Mallet III	3	

^a^Age, mean (SD): all=8.3 (2.4) years; CP=8.4 (2.7) years; group 1=7.0 (2.6) years; group 2+3=9.7 (1.5) years.

^b^GMFCS: Gross Motor Function Classification.

^c^MACS: Manual Ability Classification System.

^d^CP: cerebral palsy.

^e^N/A: not applicable.

^f^OBPP: obstetric brachial plexus palsy.

### Intervention

On average, 9.8 (range 8-11) sessions were conducted per participant, adjusting the duration of each session according to age, motivation, and fatigue. Given the fact that each player needed individual adjustments of the time limit and the objective of an exercise, comparisons could be made only through relative values; therefore, we defined 2 variables that represent the results as percentages of what was achieved in relation to what was configured: *pct_goal_* (percentage of goals, equation 1) is the number of movements performed in relation to the objective established by the therapist and *pct_time_* (percentage of time, equation 2) is the time needed to finish the exercise in relation to the time limit set.



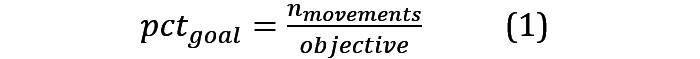





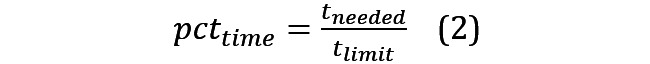



Table 3 presents the individual results that the participants obtained for each exercise during the intervention sessions.

**Figure 1:**
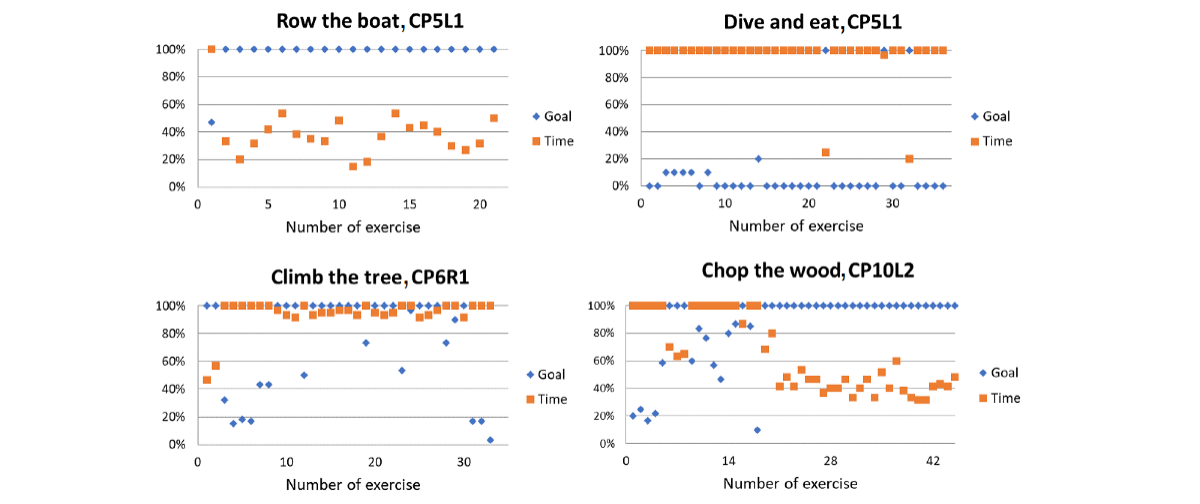
Four examples of different player experiences. Top left: easy play (100% goal achieved in a very short time). Top right: difficult play (100% time consumed but nearly no hits). Bottom left: challenged play (high percentage of goals, high time consumption). Bottom right: initially difficult play, finally easy play. The following code was used to identify the participants: CP/OBPP+age+hand+group.

For analyzing how well the target values of the exercises were adjusted in each session according to the child’s abilities, *pct_goal_* and *pct_time_* were plotted over time. The aim was to avoid excessive exertion and under exertion and to achieve the highest possible level of motivation. Figure 1 shows 4 examples of the performance of different players in different exercises, comparing the time and score achieved during the intervention.

Participant CP5L1 achieved a 100% score on the rowing exercise (Figure 1, top left) in less than half the time, and our observation was that the child quickly became bored. The plot on the right side of the top row in Figure 1 shows the opposite situation for the same child during the dive exercise: 100% of the time limit was almost always used but to no avail, and we also observed difficulties and frustration. However, in this case, it was not possible to set an easier target because it was already set at the minimum value. The third case (CP6R1 in the climbing exercise) shows a good balance between the score obtained and time taken, with both percentages being similar and mostly close to 100%. The child also played with fun and seemed challenged. The fourth case, CP10L2 in the chopping exercise (Figure 1, below right), shows a readjustment situation: the first 16 exercises were completed with a high goal and a short time limit so that the goal was almost never reached in the given time. We observed stress and reduced the goal (keeping the time limit the same), resulting in a setting that allowed reaching the goal 100% and in a short time.

**Figure 2:**
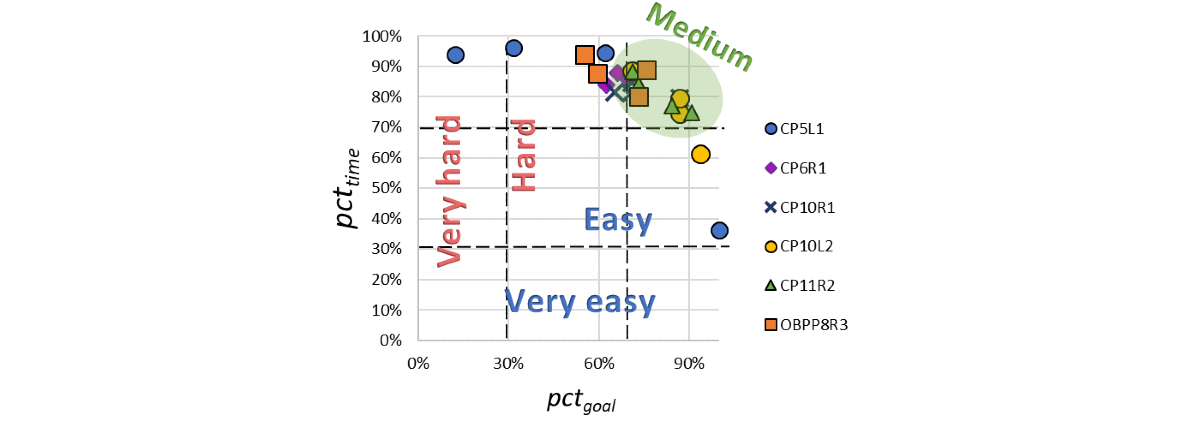
Graphical representation of all individual results for all exercises. Adjustment of time and objective. The following code was used to identify the participants: CP/OBPP+age+hand+group.

The graphical representation of all individual results from Table 3 is shown in Table 4 and in the graph in Figure 2. It shows a group of settings in the top right where both the score and the time spent are high (between 70% and 100%). Very few cases had settings that resulted in very low scores or high scores obtained in a short amount of time (blue and yellow outliers). Accordingly, and also considering our observations of the children during play, we classified the setting ranges from *very easy* to *very hard*. To do this, we divided each axis into thirds.

**Table 3:** Individual playing results, relative to the adjustments.

Participants		Chop		Row			Dive			Climb	
		*pct_goal_* ^a^_(%)_	*pct_time_* ^b^_(%)_	*pct_goal (%)_*	*pct_time (%)_*	*pct_goal (%)_*		*pct_time (%)_*	*pct_goal (%)_*		*pct_time (%)_*
**Group 1, value**											
	CP5L1	62	94	100	36	12		94	32		96
	CP6R1	74	94	92	79	22		91	66		90
	CP10R1	75	97	82	73	34		84	72		97
Group 1, mean (SD)		70 (6)	95 (1)	91 (7)	63 (19)	23 (9)		90 (4)	57 (18)		94 (3)
**Group 2+3, value**											
	CP10L2	98	52	95	75	48		93	66		97
	CP11R2	93	59	92	67	70		90	77		88
	OBPP8R3	89	85	78	75	88		78	72		81
Group 2+3, mean (SD)		93 (4)	66 (14)	88 (8)	72 (4)	69 (16)		87 (7)	72 (4)		89 (7)
All participants, mean (SD)		82 (13)	80 (18)	90 (8)	67 (14)	46 (26)		88 (6)	64 (15)		92 (6)

^a^*pct_goal_*: percentage of points obtained with respect to those established.

^b^*pct_time_*: required time percentage of set time limit.

**Table 4:** Relation between playing time, score, and adjusted game difficulty level.

*pct_time_* ^a^	*pct_goal_* ^b^	Game difficulty level
≤30%	*>pct_time_*	Very easy
>30% and ≤70%	*>pct_time_*	Easy
>70% and ≤100%	>70% and ≤100%	Medium
*>pct_goal_*	>30% and ≤70%	Hard
*>pct_goal_*	≤30%	Very hard

^a^*pct_goal_*: percentage of points obtained with respect to those established.

^b^*pct_time_*: required time percentage of set time limit.

### Data Analysis of the Exercise Results and JTHFT and BBT Assessments

**Figure 3:**
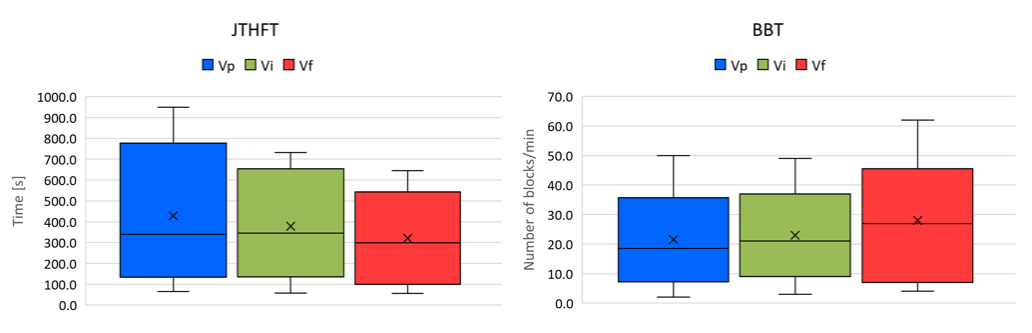
Visual comparison between the 3 evaluations with the Jebsen Taylor Hand Function Test (JTHFT) and Box and Block Test (BBT). Vf: final evaluation; Vi: initial evaluation; Vp: previous evaluation.

Table 5 presents the data of the individual results of the 3 evaluations, and Figure 3 is a visualization of these values showing the improvement in both tests.

**Figure 4:**
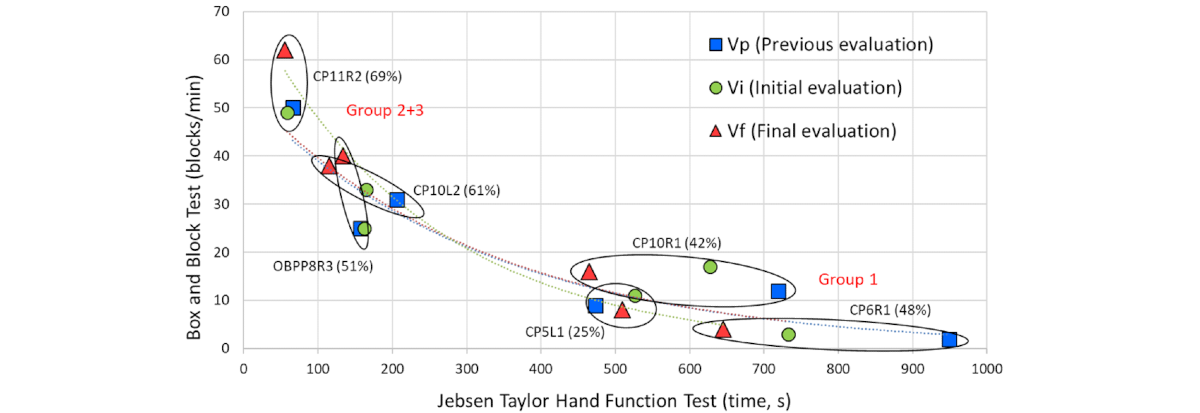
Visualization of the dependencies between evaluations, test types, and groups. Within parentheses, the percentage of time played with easy and medium adjustment of the game parameters is given. The following code was used to identify the participants: CP/OBPP+age+hand+group.

In addition, the graph in Figure 4 has been created to perform a visual analysis of the relation of the JTHFT ratings with the BBT scores for each participant. We added a hull around the 3 evaluations to show at first glance how much a child improved between assessments: the larger the shape, the more the results differed between the JTHFT and BBT. A very horizontal slant indicates better performance in the BBT, and a vertical slant indicates more improvement in the JTHFT. Round shapes indicate few differences between both assessments, and a slant toward 45° indicates that the improvements were similar.

**Table 5:** Individual results of 3 evaluations with the Jebsen Taylor Hand Function Test (JTHFT) and Box and Block Test (BBT).

Participants		JTHFT (seconds)						BBT (number of blocks)				
		V_p_^a^	V_i_^b^	V_f_^c^	V_i_ – V_p_	V_f_ – V_i_	V_p_		V_i_	V_f_	V_i_ – V_p_	V_f_ – V_i_
**Group 1, value**												
	CP5L1	473.1	526.6	509.4	53.5	−17.2	9.0		11.0	8.0	2.0	−3.0
	CP6R1	719.5	627.5	464.7	−92.0	−162.8	12.0		17.0	16.0	5.0	−1.0
	CP10R1	949.4	732.7	645.3	−216.8	−87.4	2.0		3.0	4.0	1.0	1.0
Group 1, mean (SD)		714.0 (194.49)	628.9 (84.14)	539.8 (76.81)	−85.1 (110.42)	−89.1 (59.46)	7.7 (4.19)		10.3 (5.73)	9.3 (4.99)	2.7 (1.70)	−1.0 (1.63)
**Group 2+3, value**												
	CP10L2	157.4	162.1	133.6	4.7	−28.5	25.0		25.0	40.0	0.0	15.0
	CP11R2	206.3	164.4	115.3	−41.9	−49.1	31.0		33.0	38.0	2.0	5.0
	OBPP8R3	65.9	58.4	55.5	−7.5	−3.0	50.0		49.0	62.0	−1.0	13.0
Group 2+3, mean (SD)		143.2 (58.17)	128.3 (49.42)	101.4 (33.37)	−14.9 (19.73)	−26.9 (18.87)	35.3 (10.66)		35.7 (9.98)	46.7 (10.87)	0.3 (1.25)	11.0 (4.32)
All participants, mean (SD)		428.6 (349.9)	378.6 (284.4)	320.6 (248.7)	−50.0 (95.02)	−58.0 (59.2)	21.5 (17.6)		23.0 (16.5)	28.0 (22.5)	1.5 (2.1)	5.0 (7.5)
**Between groups**												
	Difference of means	−570.83	−500.6	−438.4	−70.2	−62.3	27.66		25.34	37.4	2.33	−12
	*P* value	.02	.002	.002	.41	.23	.03		.04	.01	.19	.02

^a^V_p_: previous evaluation.

^b^V_i_: initial evaluation.

^c^V_f_: final evaluation.

In addition, we assembled the evaluation data with exponential trend lines to demonstrate the correlation between them. The resulting curves from the 2 initial assessments did not differ, indicating that the children’s mean scores were very similar. However, the curve of the third assessment stood out significantly, showing a more vertical slope, indicating that there were greater differences between JTHFT and BBT in this final assessment, pointing to an improvement in skills owing to the games.

## Discussion

The main finding of this study is the categorization of the parameter adjustments into 5 levels of difficulty, which may be of high interest to a therapist who has to decide on the configuration of different exercises. Here, the *easy* and *medium* levels seem to be the most effective, as the final assessment tests show greater improvements for those children who played longer in these adjustment ranges.

### Participants

The number of children in this study seems small at first glance, but given our conditions (being a common primary school that integrates 28 children with very different types of physical disabilities), we were fortunate to find 6 children of similar conditions and age and that they were allowed to participate in this study. However, their physical abilities were different because of different degrees of affection, so we decided to analyze them in 2 homogeneous subgroups of the same size, which turned out to be a positive decision, as can be observed from the results.

### Intervention

Table 3 presents the results of play for each child and each type of exercise as relative values between the scores and the adjustments. In this way, it was possible to quickly observe in which cases the parameter settings led to better results (the higher the percentage, the more appropriate the parameter adjustment was to the child’s capacity). The graphical representation of these values (refer to the graph in Table 4) led to a distinction of 5 levels of difficulty ranging from *very easy* to *very hard* and reveals that most exercises were played with settings that led to achievements of 70% to 100% for both values (upper right part of the graph in Table 4). This means that the objectives were achieved in most cases and that the time given was sufficient, neither too short nor too long. We also observed that in those cases, the children played with more fun and enthusiasm. In the other ranges, they performed worse and felt frustrated or bored. When the time limit was not considered a challenge, as the highest score could be achieved without much effort, the setup seemed too easy. When the goal of an exercise could not be achieved within the given time limit, the participants became stressed and frustrated, so adjustments were probably too difficult. These observations led us to consider the range between 70% and 100% as a *medium* difficulty level, which could be the optimal range of adjustment.

Figure 1 visualizes 4 typical cases showing cases of play that were too easy, too hard, and just right. Participant CP5L1 achieved a 100% score in less than half of the given time limit, so this could be considered a *very easy* adjustment. In the diving exercise, the same child needed the full amount of time given but was unsuccessful, so the configuration appeared to be *very hard*. CP6R1 achieved the score within the given time, so the configuration of this exercise was considered well suited for this child; we would call it an optimal setup. CP10L2, in the beginning, required the entire time given and achieved very low scores. After readjustment, the child never failed and quickly achieved the goal. Both situations were not ideal, too difficult at first and too easy afterward.

During the intervention, adjustments were made to the parameters of the game based on subjective observations because at that time, the therapist did not have a graphic display of the results available on the web. The adjustments made were expected to be optimal; however, subsequent analysis showed that not all children received an adequate adjustment to their abilities. For example, we noticed an incorrect setting for the participant CP5L1, both in the training sessions and in the intervention sessions, which could possibly have been avoided. Therefore, we consider it crucial that a therapist has an *objective* measure to verify whether a game setting is well adjusted for each participant. We are convinced that if we had had a tool that showed the range in which a child was playing, our adjustments would have been made more quickly and accurately.

In addition to the general results, it is worth mentioning some differences among the 4 types of exercises (or minigames) inherent in the game. Of them, 3 (75%) are aimed at arm movements (rowing, climbing, and chopping), and the child can fully concentrate on them because the avatar does not change its position when the child's position changes. Instead, the dive exercise is fully controlled in 3D; that is, each corporal movement translates into a change in the position of the avatar in the virtual space. This exercise was created to train trunk stability; however, it turned out to be the most difficult to control, especially for the youngest and most affected children. Therefore, this exercise produced frustration in some cases, especially for CP5L1. Here, we set the goals to low values, because we noticed that children avoided this exercise. They liked the chopping exercise best, which has a great animation with a sparkling axe and logs falling to the sides. Climbing and rowing were the 2 easiest exercises, and the children got much faster after learning how to do them.

### Data Analysis of the Exercise Results and JTHFT and BBT Assessments

The results obtained in the 3 evaluations of the JTHFT and BBT showed clear improvements for almost all children, especially between V_i_ and V_f_ (before and after the intervention with the video game): a reduction in time for the JTHFT and an increase in the number of blocks for the BBT (refer to the column *V_f_ – V_i_* in Table 5).

The results of group 1, which includes children with lower capacities, clearly differ from those of group 2+3, which can be observed in the difference in mean values for both groups. Group 1 had longer times for the Jebsen tests and moved fewer blocks in the BBT (3 to 5 times fewer blocks). The difference in means can be considered significant despite the small sample size; after checking for normality through Levene test, all *P* values were <.05 for the 3 assessments (JTHFT: V_p_, *P*=.02; V_i_, *P*=.002; and V_f_, *P*=.002; BBT: V_p_, *P*=.03; V_i_, *P*=.04; and V_f_, *P*=.01).

However, the improvements in both tests achieved through the intervention were different: group 1 improved their results in the JTHFT more than group 2+3. In V_f_, the average times achieved were about 5 times longer, but the improvement compared with V_i_ was 89.1 seconds, which was 3 times better than the improvement of group 2+3 (26.9 s). For the BBT, this was the other way around: after the intervention, group 2+3 achieved moving 11 blocks more than those moved before the intervention (+13%), whereas group 1 did not show any improvement caused by the game or related to the game.

An important correlation can be observed when contrasting the data obtained from the BBT and JTHFT evaluations with the time they played in the *easy* or *medium* difficulty range. As can be observed in Figure 3, the children who played most of the time with this configuration (refer to the percentages within parentheses) were the ones who achieved the best evaluations in either the BBT or JTHFT.

Looking at the individual cases, it can be observed that the improvement between the initial and final BBT ratings is best for CP11R2 (who played 69% of the time with *easy* or *medium* settings) and very high for OBPP8R3 (who played 51% of the time on *easy* or *medium*). CP10L2 played 61% of the time at those ranges and improved similarly in the BBT and JTHFT. CP10R1 (42%) and CP6R1 (48%) improved more in the JTHFT. Finally, CP5L1 unfortunately had *very hard* settings for her abilities, and as a result, she was the only child who did not improve, neither in the BBT nor in the JTHFT. As explained before, the reason was probably the young age and the general difficulty of the diving exercise, which was already set to the minimum possible goal for this child.

From these results, we understand that a correct adjustment range generates a certain challenge that leads to a longer playing time and highly motivates the player to reach the goal (a fact frequently mentioned in the literature and also called “flow” [34,35]). This is optimal from the therapeutic point of view, as overexertion and underload are avoided while achieving the highest possible level of motivation.

Comparing our proposed level adjustment in Table 4 with the generally published guidelines for game level adjustment, we observe a great coincidence. For example, Sepulveda et al [34] presented the ratio between player performance (*Cp*) and expected performance (*Ep*), as a method to measure the general performance or quality of adjustment:

Performance = Cp / Ep (3)

A value next to 1 means that the challenge is appropriate for the player, and with an error range set to 0.2, a value <0.8 indicates the game is too difficult and a value >1.2 means the game is too easy. If we apply this formula to our data, the relation *pct_goal_ / pct_time_* would be equivalent to *Cp*, with *Ep=1,* the ratio expected and desired by the therapist. With this, our *very easy* level can reach up to 3.3, *easy* up to 2.3, *medium* between 1.4 and 0.7, *hard* down to 0.4, and *very hard* down to 0.3. Therefore, compared with Sepulveda et al [34], we apply a larger and asymmetrical error margin, which is 0.3 for the harder adjustments and 0.4 for the easier adjustments. This is also graphically confirmed in the figure in Table 4, where most settings fall on the harder side. It has to be mentioned here that this classification should be confirmed or refined in more extensive follow-up studies.

Many publications can be found in the area of automatic and dynamic difficulty adjustment of game levels, as reviewed by Zohaib and Hideyuki [35]. It would be interesting to apply our ranges to one of the proposed algorithms and test whether they could be useful for an automatic adjustment of exercise games for people with disabilities. Furthermore, to the best of our knowledge, there are no publications on the adjustment of the level of play for people with motor disabilities; most approaches consider “accessibility,” for example, the studies by Bierre et al [36], Cairns et al [37], and Carr et al [38], but this is not the same. Accessibility deals with different disabilities and adapts interfaces. Making a game “easy” does not necessarily guarantee that people with disabilities can play it. Game level adjustment goes beyond accessibility, as an accessible game still needs to adapt to the players’ abilities like any other game to challenge the player; only the rules should not be the same as those for abled people.

### Limitations

As stated before, on some occasions, the Kinect has difficulty capturing the smallest students, and specific problems were detected in handling the main character of the game, which led to some frustration. We are currently developing a new version of the game based on Kinect One, which has much better detection possibilities.

Furthermore, it is necessary to perform tests with larger population groups and analyze the changes that would appear at a longer intervention time. For future studies, it is important to assess whether benefits are maintained in the long term and analyze motivation. Currently, we are conducting a long-term study on the same game but using an improved web interface that visualizes the range in which the users are playing. In this way, readjustments can be made according to an objective measure and, if necessary, after each session.

### Conclusions

The use of video games is considered a viable intervention to carry out in physical therapy sessions in the school environment. We observed that when the parameters were not well adjusted, the play time was short, which led to less changes in motor function (proved with the BBT and JTHFT evaluations).

The adjustment of the game seems to be related to the motor benefits found, so it would be appropriate to have access to the patients’ game performance data easily and continuously to be able to modify the parameters in time and to work within the adjusted range. Therefore, we consider it a good option to use the criteria established in this study to achieve an adequate adjustment, seeking that the child performs most of the game in the range between 70% and 100% of the goal and time. However, exhaustive tests must be performed now to prove these values.

## Abbreviations

BBT: Box and Block Test
CEIP: Centro de Educación Infantil y Primaria
CP: cerebral palsy
GMFCS: Gross Motor Function Classification
ICF: International Classification of Functioning, Disability, and Health
JTHFT: Jebsen Taylor Hand Function Test
MACS: Manual Ability Classification System
OBPP: obstetric brachial plexus palsy
UPM: Universidad Politécnica de Madrid
Vf: final evaluation
Vi: initial evaluation
Vp: previous evaluation
VR: virtual reality
